# EGCG Suppresses ERK5 Activation to Reverse Tobacco Smoke-Triggered Gastric Epithelial-Mesenchymal Transition in BALB/c Mice

**DOI:** 10.3390/nu8070380

**Published:** 2016-07-20

**Authors:** Ling Lu, Jia Chen, Hua Tang, Ling Bai, Chun Lu, Kehuan Wang, Manli Li, Yinmei Yan, Ling Tang, Rui Wu, Yang Ye, Longtao Jin, Zhaofeng Liang

**Affiliations:** 1 Zhenjiang Matemity and Child Health Care Hospital, Zhenjiang 212001, China; lu_ling60@sina.com (L.L.); lengyuing@163.com (J.C.); zjfyeb@163.com (H.T.); bai_ling1234@126.com (L.B.); lucn816@163.com (C.L.); m17705286156@163.com (K.W.); limanli123@126.com (M.L.); 18752970933@163.com (Y.Y.); m17705286167_1@163.com (L.T.); w871647946@163.com (R.W.); 2 Department of Preventive Medicine and Public Health Laboratory Sciences, School of Medicine, Jiangsu University, Zhenjiang 212013, China; jamesnone@163.com; 3 Jiangsu Key Laboratory of Medical Science and Laboratory Medicine, School of Medicine, Jiangsu University, Zhenjiang 212013, China

**Keywords:** tobacco smoke, gastric cancer, ERK5, epithelial-mesenchymal transition, EGCG

## Abstract

Tobacco smoke is an important risk factor of gastric cancer. Epithelial-mesenchymal transition is a crucial pathophysiological process in cancer development. ERK5 regulation of epithelial-mesenchymal transition may be sensitive to cell types and/or the cellular microenvironment and its role in the epithelial-mesenchymal transition process remain elusive. Epigallocatechin-3-gallate (EGCG) is a promising chemopreventive agent for several types of cancers. In the present study we investigated the regulatory role of ERK5 in tobacco smoke-induced epithelial-mesenchymal transition in the stomach of mice and the preventive effect of EGCG. Exposure of mice to tobacco smoke for 12 weeks reduced expression of epithelial markers E-cadherin, ZO-1, and CK5, while the expression of mesenchymal markers Snail-1, Vimentin, and *N*-cadherin were increased. Importantly, we demonstrated that ERK5 modulated tobacco smoke-mediated epithelial-mesenchymal transition in mice stomach, as evidenced by the findings that tobacco smoke elevated ERK5 activation, and that tobacco smoke-triggered epithelial-mesenchymal transition was reversed by ERK5 inhibition. Treatment of EGCG (100 mg/kg BW) effectively attenuated tobacco smoke-triggered activation of ERK5 and epithelial-mesenchymal transition alterations in mice stomach. Collectively, these data suggested that ERK5 was required for tobacco smoke-triggered gastric epithelial-mesenchymal transition and that EGCG suppressed ERK5 activation to reverse tobacco smoke-triggered gastric epithelial-mesenchymal transition in BALB/c mice. These findings provide new insights into the mechanism of tobacco smoke-associated gastric tumorigenesis and the chemoprevention of tobacco smoke-associated gastric cancer.

## 1. Introduction

Gastric cancer is the fourth most common cancer and the second leading cause of cancer deaths worldwide [1]. In 2012, a total of 951,600 new gastric cancer cases and 723,100 deaths are estimated to have occurred, accounting for 8% of the total cases and 10% of total deaths of cancer [2]. In China, the incidence of gastric cancer ranks third in all malignant tumors, with an estimated 380,000 new cases every year, and the mortality rate for gastric cancer is approximately 17.85 per 100,000 [3,4,5].

Many factors are associated with the initiation and development of gastric cancer, including genetic variations, dietary factors, environmental factors, infectious agents, and pathological conditions in the stomach [6]. Tobacco smoke is an important risk factor for gastric cancer. Studies have revealed the positive link between tobacco smoke and risk of gastric cancer initiation and development [7,8,9,10,11,12]. Many ingredients found in tobacco smoke are known to induce free radicals and possess toxic properties, thereby contributing to its carcinogenic potential, including its potential impact on the transformation and progression of cancer [8,13]. Although enormous progress in understanding its molecular mechanisms leading to gastric cancer initiation and development has been made, the molecular pathogenesis is not fully understood.

Epithelial-mesenchymal transition is an important pathophysiological process in embryonic progression as well as cancer development [14,15]. Evidence has indicated that epithelial-mesenchymal transition is critically involved in the initiation of cancer by promoting cell malignant transformation. During the epithelial-mesenchymal transition process, cells progressively lose epithelial characteristics and acquire mesenchymal features. Tobacco smoke has been shown to promote the epithelial-mesenchymal transition process [16,17,18]. Tobacco smoke-induced epithelial-mesenchymal transition has been found to regulate the early events in cancer oncogenesis including down-regulation of epithelial cadherin, loss of cell-cell adhesion, and increased mobility of cells. However, the mechanism regarding how tobacco smoke mediates epithelial-mesenchymal transition changes and the signaling events that underlie epithelial-mesenchymal transition remains largely unknown.

ERK5, also termed big MAPK1, is the least studied member of mitogen-activated protein kinase (MAPK) family, and is implicated in important cellular processes: gene expression, proliferation, apoptosis, angiogenesis, cell motility, and differentiation [19,20,21,22]. Some studies have suggested the functions of ERK5 in carcinogenesis; nonetheless, its role in epithelial-mesenchymal transition regulation has not been well explored. It has been reported that ERK5 promote epithelial-mesenchymal transition [23,24,25]. On the contrary, several studies have suggested activation of ERK5 suppresses epithelial-mesenchymal transition [26,27]. To date, no studies have been done to examine the role of ERK5 in tobacco smoke-induced gastric epithelial-mesenchymal transition.

Epigallocatechin-3-gallate (EGCG), the most abundant and active polyphenol in green tea, has been shown to have anti-inflammatory, anti-oxidant, anti-cancer, and chemopreventive properties [28,29,30]. Previously obtained evidence indicates that EGCG has a therapeutic potential in preventing and treating several chronic diseases, including various types of cancer [29,30,31,32]. However, its effect on tobacco smoke-induced gastric epithelial-mesenchymal transition has not been defined.

Our present study was designed to investigate ERK5 regulation of tobacco smoke-induced epithelial-mesenchymal transition and the preventive effects of EGCG against tobacco smoke-induced epithelial-mesenchymal transition alterations in the stomach of mice. By using *in vivo* tobacco smoke exposure models, we demonstrate that ERK5 regulated tobacco smoke-mediated gastric epithelial-mesenchymal transition and the protective effects of EGCG in tobacco smoke-induced ERK5 activation and epithelial-mesenchymal transition in the stomach tissue of mice for the first time. These novel findings suggest the important role of ERK5 in tobacco smoke-associated gastric carcinogenesis and open new avenues in search for potential interventional targets of tobacco smoke-associated gastric cancer.

## 2. Experimental Section

### 2.1. Chemicals and Reagents

The primary antibodies phosphorylated ERK5, phosphorylated c-Fos, E-cadherin, CK5, Snail-1, *N*-cadherin, and Vimentin were obtained from Cell Signaling Technology (Beverly, MA, USA). The antibody for ZO-1 was from Santa Cruz Biotechnology (Santa Cruz, CA, USA). The glyceraldehyde-3-phosphate gehydrogenase (GAPDH) antibody was from Biogot Technology (Nanjing, China). EGCG was purchased from Sigma (St. Louis, MO, USA, purity: 98%). XMD8-92 was purchased from Tocris Bioscience (Bristol, UK). The primers were synthesized according to published sequences from Invitrogen (Carlsbad, CA, USA). Sources of other materials are noted accordingly in the text.

### 2.2. Mice and Tobacco Smoke Exposure

Eight-week-old BALB/c mice weighing 18–22 g were purchased from the Animal Research Center of Nanjing Medical University. Mice were group-housed in polypropylene cages, maintained on a 12-h light/dark cycle, 22 °C–25 °C room temperature and 50%–65% relative humidity. Basal food in pellet form and tap water were available *ad libitum* at all times during the study. Mice were handled in accordance with the recommendations in the guidelines of the Animal Care and Welfare Committee of Jiangsu University. The study protocol was approved by the Committee on the Ethics of Animal Experiments of Jiangsu University.

Six mice were randomly assigned into each group. Mice in the control group were exposed to filtered air. The tobacco smoke exposure group was exposed to tobacco smoke in a smoking device. smoke was drawn out of one commercial cigarette (Hongtashan, one of the most consumed cigarettes in China, contains 12 mg tar and 1.1 mg nicotine per cigarette) with a vacuum, which smoked the cigarettes and pumped the mainstream cigarette smoke from burning cigarettes at a constant rate (each cigarette took 5 min to burn out). Smoke was delivered to whole-body exposure chambers with a target concentration of total particulate matter (TPM) of 80 mg/m^3^. Mice were exposed for 6 h daily for 12 weeks. The exposures were monitored and characterized as the following: carbon monoxide (13.23 ± 2.72 mg/m^3^), TPM (0 mg/m^3^) for the control group; carbon monoxide (157.56 ± 20.12 mg/m^3^), TPM (79.73 ± 3.92 mg/m^3^) for the tobacco smoke exposure group. Following the completion of the exposure, mice were sacrificed and the stomach tissues were isolated for analysis.

### 2.3. In Vivo Delivery of Specific ERK5 Inhibitor

Mice were randomly divided into four groups (*n* = 10 per group): filtered air group, mice were exposed to filtered air; tobacco smoke-exposed group, mice were exposed to tobacco smoke; tobacco smoke + dimethyl sulfoxide (DMSO) group, mice were injected with DMSO and exposed to tobacco smoke; tobacco smoke + XMD8-92 group, mice were injected with XMD8-92 and exposed to tobacco smoke. XMD8-92, a highly specific ERK5 inhibitor was reconstituted in sterile DMSO and injected intraperitoneally (2 mg/kg body weight) every other day. Mice were weighed weekly. After the last tobacco smoke exposure, mice were sacrificed and stomach tissues were collected, frozen, and stored at −80 °C until analysis.

### 2.4. EGCG Treatment of Mice

In a separate set of animal studies, mice were treated daily with EGCG (50 or 100 mg/kg body weight (BW) per day, p.o.). Mice were divided into four groups (*n* = 10 per group): filtered air group, mice were exposed to filtered air and received control diet (AIN-76A); tobacco smoke-exposed group, mice were exposed to tobacco smoke and received control diet; tobacco smoke + EGCG 50 mg/kg, mice were exposed to tobacco smoke and received control diet supplemented with EGCG at a dose of 50 mg/kg BW/day; tobacco smoke + EGCG 100 mg/kg, mice were treated with 100 mg/kg BW/day EGCG and exposed to tobacco smoke. Animals were weighed weekly. The administration dosages of EGCG were based on the measurements of mouse body weight and the amount of diet consumption. After the last tobacco smoke exposure, mice were sacrificed and stomach tissues were collected, frozen, and stored at −80 °C until analysis.

### 2.5. Western Blot Analysis

Gastric tissues were homogenized in a lysate buffer and then centrifuged at 4 °C for 25 min. Protein concentrations were measured and sixty micrograms of proteins were fractionated by electrophoresis through 7.5% or 10% sodium dodecyl sulfate polyacrylamide gel electrophoresis (SDS-PAGE) and then transferred to polyvinylidene fluoride (PVDF) membrane (Millipore, Billerica, MA, USA). The membranes were blocked with 5% defatted milk and subsequently probed with primary antibodies overnight at 4 °C, and then incubated with horseradish peroxidase-conjugated secondary antibody. For densitometric analyses, protein bands on the blots were measured by the use of Eagle Eye II software.

### 2.6. Quantitative Real-Time PCR

The RNA of the stomach tissues was isolated by RNAiso Plus according to the manufacturer’s instructions (TaKaRa Biotechnology, Dalian, China). Realtime quantitative PCR (QRT-PCR) was performed by using the Power SYBR Green Master Mix (TaKaRa Biotechnology, Dalian, China) and an ABI 7300 real-time PCR detection system (Applied Biosystems, Foster, CA, USA). The primers used were as follows: E-cadherin, forward 5′-TCGACACCCGATTCAAAGTGG-3′ and reverse 5′-TTCCAGAAACGGAGGCCTGAT-3′; ZO-1, forward 5′-GCAGCCACAACCAATTCATAG-3′ and reverse 5′-GCAGACGATGTTCATAGTTTC-3′; CK5, forward 5′-CTGGAGAGTAGTCTAGACCAAGCC-3′ and reverse 5′-GTTAGAACCAAAACAAAATTTGGG-3′; Snail-1, forward 5′-GACCACTATGCCGCGCTCTT-3′and reverse 5′-TCGCTGTAGTTAGGCTTCCGATT-3′; Vimentin, forward 5′-CCTTGACATTGAGATTGCCA-3′ and reverse 5′-GTATCAACCAGAGGGAGTGA-3′; *N*-cadherin forward 5′-ATCAAGTGCCATTAGCCAAG-3′ and reverse 5′-CTGAGCAGTGAATGTTGTCA-3′; GAPDH, forward 5′-GCTGCCCAACGCACCGAATA-3′ and reverse 5′-GAGTCAACGGATTTGGTCGT-3′. Fold changes in gene expression were calculated by a comparative threshold cycle (Ct) method using the formula 2^−ΔΔCt^.

### 2.7. Immunohistochemistry

After the last tobacco smoke exposure, mice were sacrificed and stomach tissues were collected. Immunohistochemistry was performed according to the reported method. Briefly, 5 μm serial coronal gastric sections (paraffin-embedded) were de-waxed in xylene and rehydrated in graded alcohol, after which endogenous peroxidase activity was quenched by incubating the sections in 3% (*v*/*v*) H_2_O_2_ in methanol. Antigen-retrieval was performed by incubating the sections in citrate buffer (pH 6.0). Non-specific binding was blocked by 5% bovine serum albumin. After overnight incubation with the primary antibody (E-cadherin and Vimentin) at 4 °C, the sections were subsequently washed with phosphate buffer solution (PBS) before incubation for 1 h with biotinylated goat anti-rabbit immunoglobulin G (IgG) diluted 1:200 in PBS. Finally, the slices were mounted with neutral gum for microscopic examination, and cells with brown granules in the cytoplasm or nucleolus were considered positive. Images were collected using a Nikon eclipse Ti-S microscope at a 200× magnification.

### 2.8. Statistical Analysis

Statistical analyses were performed with SPSS 18.0. All data were expressed as mean ± standard deviation. One-way ANOVA was used for comparison of statistical differences among multiple groups, followed by the multiple comparison (LSD) significant difference tests. Unpaired Student’s test was also used for the comparison between two groups. A value of *p* < 0.05 was considered significantly different.

## 3. Results

### 3.1. Tobacco Smoke Induced Epithelial-Mesenchymal Transition Changes in Gastric Tissues of Mice

**Figure 1:**
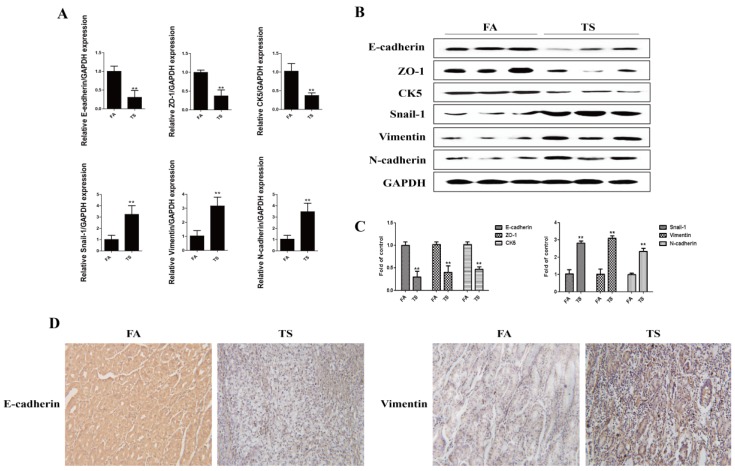
Tobacco smoke induced alterations in the expression of epithelial-mesenchymal transition markers. (**A**) Tobacco smoke reduced mRNA levels of E-cadherin, ZO-1, and CK5, and elevated mRNA levels of Snial-1, Vimentin, and *N*-cadherin in the stomach of mice exposed to tobacco smoke for 12 weeks; (**B**) Tobacco smoke decreased the protein levels of E-cadherin, ZO-1, and CK5, and increased the protein levels of Snial-1, Vimentin, and *N*-cadherin in the stomachs of mice; (**C**) Densitometric analyses of Western blotting; (**D**) Tobacco smoke decreased E-cadherin protein expression and increased Vimentin protein expression shown by immunohistochemical staining. Data are expressed as mean ± SD. ** *p* < 0.01, compared with control. FA = filtered air; TS = tobacco smoke.

Tobacco smoke is one of the primary risk factors for gastric cancer. Tobacco smoke-triggered epithelial-mesenchymal transition is critically involved in tobacco smoke-associated malignant transformation. In the present study, we investigated whether tobacco smoke induces epithelial-mesenchymal transition in gastric tissues. Mice were exposed to tobacco smoke for 12 weeks, and then the mRNA and protein expression levels of the epithelial markers and the mesenchymal markers in the stomach of mice were examined. Results showed that tobacco smoke exposure decreased the mRNA expression of E-cadherin, ZO-1, and CK5, and increased the mRNA expression levels of Snial-1, Vimentin, and *N*-cadherin (Figure 1A). Furthermore, tobacco smoke exposure reduced E-cadherin, ZO-1, and CK5 protein expression, and elevated Snial-1, Vimentin, and *N*-cadherin protein levels (Figure 1B,C). Immunohistochemical staining also showed that tobacco smoke decreased E-cadherin protein expression and increased Vimentin protein expression in the stomach of mice (Figure 1D).

### 3.2. Tobacco Smoke-Induced Gastric Epithelial-Mesenchymal Transition Was Associated with ERK5 Activation

**Figure 2:**
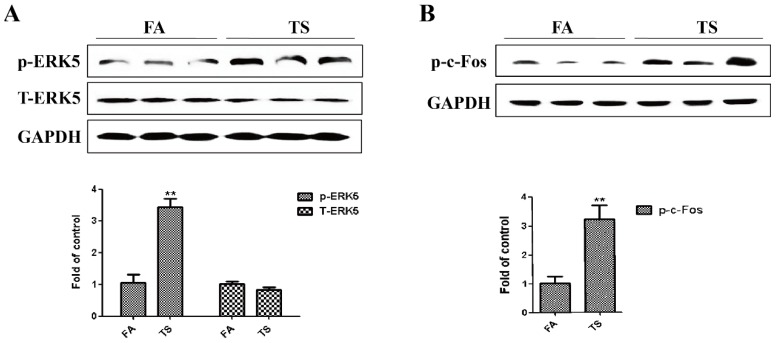
Tobacco smoke increased ERK5 activation in the stomach tissues of mice. (**A**) Western blotting analyses of phosphorylated ERK5 and total ERK5 in the stomach of mice exposed to tobacco smoke for 12 weeks; (**B**) Western blotting analyses of phosphorylated c-Fos in the stomach of mice. Data are expressed as mean ± SD. ** *p* < 0.01, compared with control. FA = filtered air; TS = tobacco smoke.

As the least studied member of the MAPK family, ERK5 is implicated in carcinogenesis. The action of ERK5 in epithelial-mesenchymal transition regulation has not been well explored, although evidence has suggested that a differential regulatory role of ERK5 on epithelial-mesenchymal transition may exist. To determine whether tobacco smoke-induced gastric epithelial-mesenchymal transition is associated with ERK5 activation, the expression level of phosphorylated ERK5, the indicator of ERK5 activation status, was measured. It was found that tobacco smoke exposure activated gastric ERK5 (Figure 2A). We also found that tobacco smoke elevated levels of phosphorylated c-Fos (Figure 2B).

### 3.3. ERK5 Suppression Reversed Tobacco Smoke-Triggered Gastric Epithelial-Mesenchymal Transition

**Figure 3:**
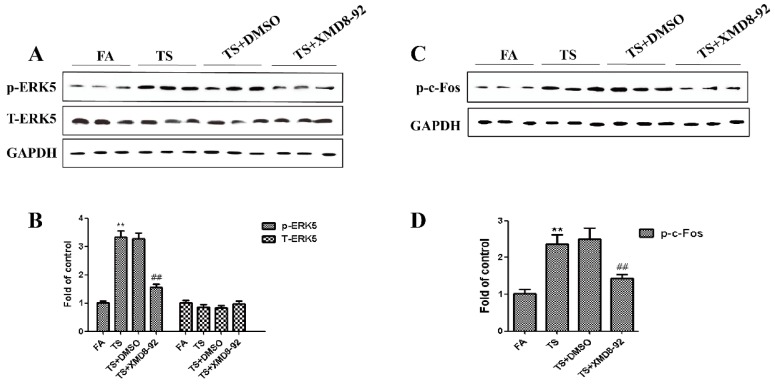
Tobacco smoke-induced ERK5 activation was attenuated by XMD8-92 in the stomach of mice. (**A**) Western blotting analyses of phosphorylated ERK5 and total ERK5; (**B**) Densitometric analyses of Western blotting; (**C**) Western blotting analyses of phosphorylated c-Fos, phosphorylated c-Jun; (**D**) Densitometric analyses of Western blotting. Data are expressed as mean ± SD. ** *p* < 0.01, compared with control; ^##^ FA = filtered air; TS = tobacco smoke.

**Figure 4:**
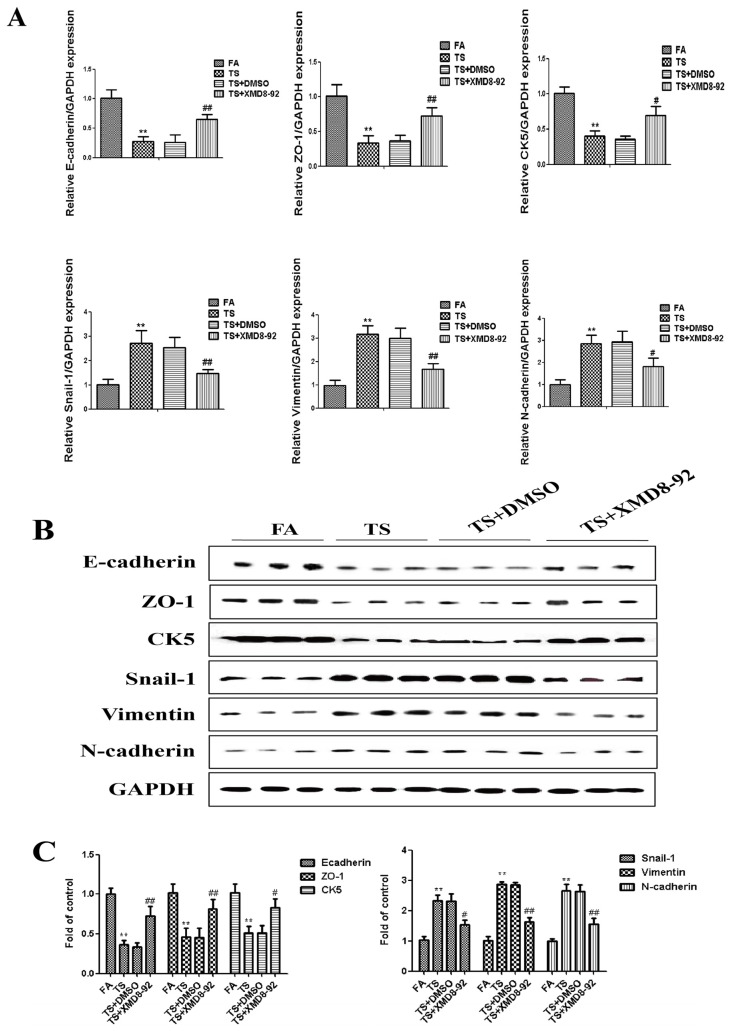
ERK5 suppression reversed tobacco smoke-induced gastric epithelial-mesenchymal transition alterations in mice. (**A**) qRT-PCR analyses of E-cadherin, ZO-1, CK5, Snial-1, Vimentin, and *N*-cadherin mRNAs; (**B**) Western blotting analyses of E-cadherin, ZO-1, CK5, Snial-1, Vimentin, and *N*-cadherin proteins; (**C**) Densitometric analyses of Western blotting. Data are expressed as mean ± SD. ** *p* < 0.01, compared with FA control; ^#^ *p* < 0.05, compared with TS; ^#^ *p* < 0.05, ^##^ *p* < 0.01, compared with TS. FA = filtered air; TS = tobacco smoke.

Since above results revealed that tobacco smoke-induced gastric epithelial-mesenchymal transition was associated with the change of ERK5 activation, we further determined the role of ERK5 in gastric epithelial-mesenchymal transition regulation. Mice were treated with XMD8-92 (2 mg/kg body weight), a highly specific ERK5 inhibitor. Results showed that XMD8-92 down-regulated phosphorylated ERK5 and AP-1 expression levels (Figure 3A–D). Tobacco smoke-induced alterations in the mRNAs of E-cadherin, ZO-1, CK5, Snial-1, Vimentin, and *N*-cadherin were effectively attenuated by XMD8-92 (Figure 4A). Western blot analyses further showed that treated mice with XMD8-92 reversed tobacco smoke-induced expression change of E-cadherin, ZO-1, CK5, Snial-1, Vimentin, and *N*-cadherin (Figure 4B,C).

### 3.4. EGCG Attenuated Tobacco Smoke- Induced Gastric Epithelial-Mesenchymal Transition in the Stomach of Mice

**Figure 5:**
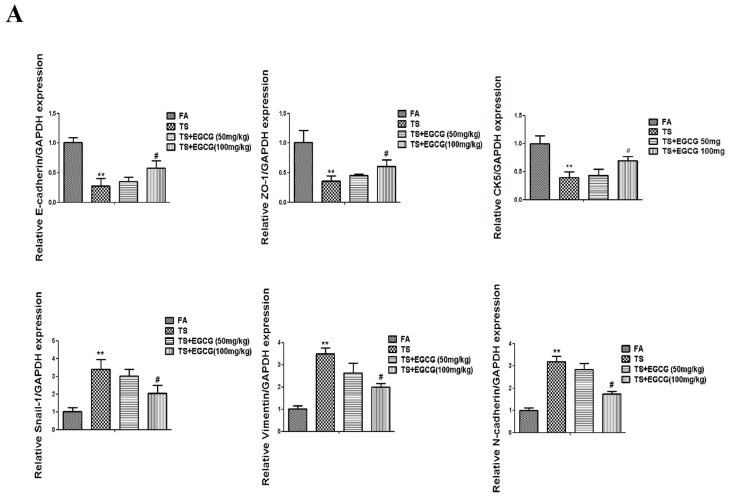

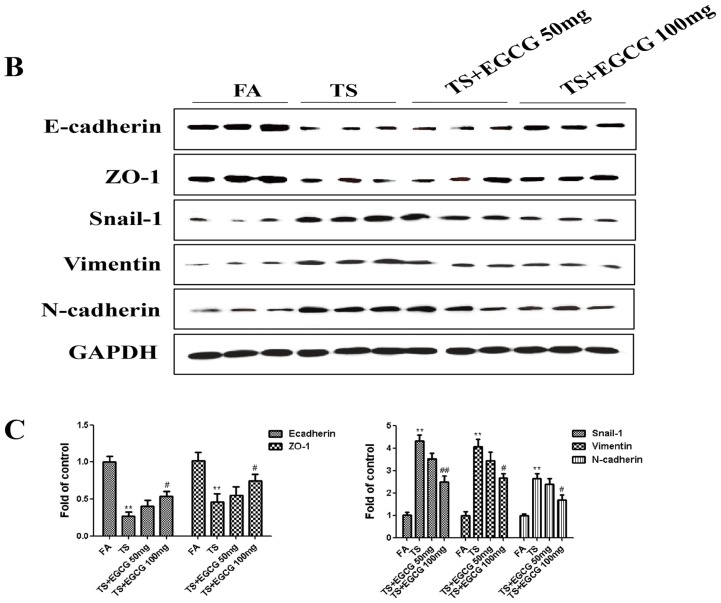
EGCG attenuated tobacco smoke-induced alterations of epithelial-mesenchymal transition markers in the stomach of mice. (**A**) qRT-PCR analyses of E-cadherin, ZO-1, CK5, Snail-1, Vimentin, and *N*-cadherin mRNAs; (**B**) Western blotting analyses of E-cadherin, ZO-1, Snail-1, Vimentin, and *N*-cadherin proteins; (**C**) Densitometric analyses of Western blotting. Data are expressed as mean ± SD. ** *p* < 0.01, compared with FA control; ^#^ *p* < 0.05, compared with TS; ^##^ *p* < 0.01, compared with TS. FA = filtered air; TS = tobacco smoke.

In order to determine the effects of EGCG on tobacco smoke-mediated epithelial-mesenchymal transition in the stomach, mice received EGCG (50 or 100 mg/kg BW/day) and were exposed to tobacco smoke for 12 weeks. Figure 5 shows that tobacco smoke-induced alterations in mRNA and protein expressions of the epithelial-mesenchymal transition markers—including decreases of the epithelial markers E-cadherin, ZO-1, CK5, and increases of the mesenchymal markers Snail-1, Vimentin, *N*-cadherin—were effectively attenuated with 100 mg/kg BW/day EGCG treatment. These data indicated that EGCG attenuated tobacco smoke-induced gastric epithelial-mesenchymal transition *in vivo*. 

### 3.5. EGCG Reversed Tobacco Smoke-Induced ERK5 Activation in the Stomach of Mice

**Figure 6:**
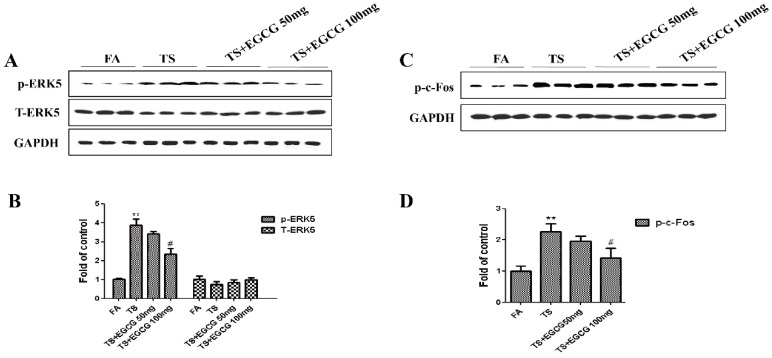
EGCG suppressed tobacco smoke-induced ERK5 activation in mice stomach. (**A**) Western blotting analyses of phosphorylated ERK5 and total ERK5; (**B**) Densitometric analyses of Western blotting; (**C**) Western blotting analyses of phosphorylated c-Fos; (**D**) Densitometric analyses of Western blotting. Data are expressed as mean ± SD. ** *p* < 0.01, compared with FA control; ^#^ *p* < 0.05, compared with TS. FA = filtered air; TS = tobacco smoke.

To explore the influence of EGCG on tobacco smoke-mediated activation of EKR5, we further examined the change of ERK5 activation following EGCG treatment in the stomach of mice. Western blot analyses showed that 100 mg/kg BW/day EGCG significantly inhibited tobacco smoke-induced ERK5 activation (Figure 6A,B). Treatment of EGCG also decreased tobacco smoke-induced c-Fos (Figure 6C,D).

## 4. Discussion

Gastric cancer is still one of the leading causes of cancer mortality worldwide. The relationship between the occurrence of gastric cancer and tobacco smoke has been established. Tobacco smoke is one of the leading causes of gastric cancer, which promotes the initiation and progression of gastric tumorigenesis. However, the underlying molecular mechanisms by which tobacco smoke causes the development of gastric cancer remain to be established. In the present study we revealed that tobacco smoke induced epithelial-mesenchymal transition changes in the stomach of mice. Most importantly, we demonstrated for the first time that ERK5 regulated tobacco smoke-mediated gastric epithelial-mesenchymal transition, and that tobacco smoke-induced ERK5 activation and tobacco smoke-induced gastric epithelial-mesenchymal transition were reversed by EGCG *in vivo*.

Characterized by changes in migration and invasion capacity, as well as the expression of epithelial and mesenchymal markers, epithelial-mesenchymal transition is a crucial process in cancer initiation and development [14,15]. It has been documented that tobacco smoke promotes the epithelial-mesenchymal transition process, resulting in loss of cellular polarity, down-regulation of epithelial cadherin, the acquisition of mesenchymal features, and increased mobility of cells [16,17,18]. Consistent with previous reports, we found that long-term exposure to tobacco smoke induced epithelial-mesenchymal transition in the stomach of mice. Tobacco smoke altered the expression of epithelial-mesenchymal transition markers, including decreased epithelial markers E-cadherin, ZO-1, and CK5, and increased mesenchymal markers Snail-1, Vimentin, and *N*-cadherin. These results revealed that long-term tobacco smoke exposure triggered gastric epithelial-mesenchymal transition *in vivo*.

Nonetheless, the underlying mechanisms of epithelial-mesenchymal transition induction by tobacco smoke are poorly understood. As the proto-oncogenic signaling, MAPKs pathways have been reported to promote epithelial-mesenchymal transition. Our previous study had suggested the role of ERK1/2 and JNK MAPK pathways in the curcumin-mediated protective effect against tobacco smoke-elicited gastric epithelial-mesenchymal transition changes [6]. However, differential biological functions may exist for individual MAPK pathways. Among the MAPK family, ERK5 is twice the size of other members and is the lesser studied member. Unlike other MAPK members, the C-terminal region of ERK5 contains a long non-catalytic domain which has a unique function. Upon activation, ERK5 phosphorylates and activates downstream target molecules, including transcription factors such as members of the AP-1 proteins. Activation of ERK5 also results in autophosphorylation of the C-terminus, which alone has the ability to increase transcription activity. Therefore, ERK5 differs from other MAPKs in possessing transcriptional activation activity. Some reports have depicted the important biological functions of ERK5 in cancer oncogenesis; however, its role in epithelial-mesenchymal transition regulation has not been well characterized. It has been reported that ERK5 promotes epithelial-mesenchymal transition [23,24,25]. On the other hand, evidence has suggested a differential regulatory role of ERK5 on epithelial-mesenchymal transition. ERK5 activation results in the significantly decreased migration and invasion of breast cancer cells [26] and negatively regulates hepatic and endothelial cell migration [33,34,35]. Our previous study also demonstrated that ERK5 negatively regulates tobacco smoke-induced pulmonary epithelial-mesenchymal transition in both *in vitro* and *in vivo* settings [36]. This evidence suggested that the action of ERK5 in epithelial-mesenchymal transition process may be sensitive to cell types and/or the cellular microenvironment.

In the present study we showed that tobacco smoke-induced gastric epithelial-mesenchymal transition was associated with upregulation of ERK5 activation *in vivo*. To determine the role of ERK5 in gastric epithelial-mesenchymal transition regulation, mice were treated with XMD8-92 (0.5 mg/kg body weight), a highly specific ERK5 inhibitor which suppresses ERK5 autophosphorylation. As expected, XMD8-92 down-regulated phosphorylated ERK5 levels as well as levels of phosphorylated c-Fos. Furthermore, inhibition of ERK5 attenuated tobacco smoke-induced alterations in epithelial-mesenchymal transition markers, including decreased epithelial markers E-cadherin, ZO-1, and CK5, and increased mesenchymal markers Snail-1, Vimentin, and *N*-cadherin. These data clearly indicated for the first time that ERK5 positively regulates tobacco smoke-induced gastric epithelial-mesenchymal transition *in vivo* setting.

Chemoprevention has been shown to be a rational and very promising approach to the prevention of cancer development, especially in high-risk populations. As a dietary polyphenol, EGCG is widely used in healthcare, food, and cosmetics products which possess various biological activities and excellent tolerance [37,38]. Some studies have reported the safety of EGCG as well as its anticancer activities in relation to many cancers [28,39]. The concentrations of EGCG used in the present study were 50 and 100 mg/kg BW per day. The doses of EGCG are equivalent to 3–6 mg/kg for a human [40]. These doses of EGCG can be achieved by consuming 2–4 cups of green tea. After treatment with 50 or 100 mg/kg BW/day doses of EGCG, the effect of EGCG on tobacco smoke-triggered alterations in the mRNA and protein expression of the epithelial-mesenchymal transition markers were examined. Results showed that tobacco smoke-induced gastric epithelial-mesenchymal transition alterations were attenuated by 100 mg/kg BW/day doses of EGCG. We further revealed that the administration of EGCG at 100 mg/kg BW/day doses suppressed tobacco smoke-mediated activation of ERK5. Tobacco smoke-elevated level of phosphorylated c-Fos was also reduced following the 12-week EGCG treatment. Collectively, these data suggest the protective effect of EGCG against tobacco smoke-elicited gastric epithelial-mesenchymal transition changes *in vivo.*

## 5. Conclusions

Our present study demonstrated for the first time that ERK5 positively regulates tobacco smoke-induced gastric epithelial-mesenchymal transition and the protective effects of EGCG in tobacco smoke-induced ERK5 activation and epithelial-mesenchymal transition *in vivo*. These findings provide new insight into the mechanisms of tobacco smoke-associated gastric tumorigenesis and the chemoprevention of tobacco smoke-associated gastric cancer.
